# Do patients prefer a human doctor, artificial intelligence, or a blend, and is this preference dependent on medical discipline? Empirical evidence and implications for medical practice

**DOI:** 10.3389/fpsyg.2024.1422177

**Published:** 2024-08-12

**Authors:** René Riedl, Svea A. Hogeterp, Martin Reuter

**Affiliations:** ^1^Digital Business Institute, University of Applied Sciences Upper Austria, Steyr, Austria; ^2^Institute of Business Informatics – Information Engineering, University of Linz, Linz, Austria; ^3^Institute of Psychology, University of Bonn, Bonn, Germany

**Keywords:** artificial intelligence, chatbot, trust, doctor-patient relationship, medicine, patient-doctor interaction, privacy invasion, psychiatry

## Abstract

Today the doctor-patient relationship typically takes place in a face-to-face setting. However, with the advent of artificial intelligence (AI) systems, two further interaction scenarios are possible: an AI system supports the doctor’s decision regarding diagnosis and/or treatment while interacting with the patient, or an AI system could even substitute the doctor and hence a patient interacts with a chatbot (i.e., a machine) alone. Against this background, we report on an online experiment in which we analyzed data from *N* = 1,183 people. The data was collected in German-speaking countries (Germany, Austria, Switzerland). The participants were asked to imagine they had been suffering from medical conditions of unknown origin for some time and that they were therefore visiting a health center to seek advice from a doctor. We developed descriptions of patient-doctor interactions (referred to as vignettes), thereby manipulating the patient’s interaction partner: (i) human doctor, (ii) human doctor with an AI system, and (iii) an AI system only (i.e., chatbot). Furthermore, we manipulated medical discipline: (i) cardiology, (ii) orthopedics, (iii) dermatology, and (iv) psychiatry. Based on this 3 × 4 experimental within-subjects design, our results indicate that people prefer a human doctor, followed by a human doctor with an AI system, and an AI system alone came in last place. Specifically, based on these 12 hypothetical interaction situations, we found a significant main effect of a patient’s interaction partner on trust, distrust, perceived privacy invasion, information disclosure, treatment adherence, and satisfaction. Moreover, perceptions of trust, distrust, and privacy invasion predicted information disclosure, treatment adherence, and satisfaction as a function of interaction partner and medical discipline. We found that the situation in psychiatry is different from the other three disciplines. Specifically, the six outcome variables differed strongly between psychiatry and the three other disciplines in the “human doctor with an AI system” condition, while this effect was not that strong in the other conditions (human doctor, chatbot). These findings have important implications for the use of AI in medical care and in the interaction between patients and their doctors.

## 1 Introduction

The *relationship* between doctor and patient has been crucial since the early days of ancient civilizations, because it affects major medical outcomes such as patient information disclosure, treatment adherence, satisfaction, and ultimately treatment success (e.g., McGrew, 1985; Stewart, 1995; Elgood, 2010; Spikins et al., 2018). What has characterized the doctor-patient relationship in the past and still does today is that the interaction typically takes place face-to-face. However, due to technological advancements, changes in this mode of interaction have become evident. Telemedicine (defined as distribution of health-related services and information via information and communication technologies, ICT) has become, at least in some contexts, an accepted practice (e.g., Hollander and Carr, 2020; Mann et al., 2020). Example contexts are the provision of medical services in rural settings or if social distancing is required such as in the case of infectious diseases like COVID-19. Telemedicine, however, constitutes computer-mediated communication. Thus, ICT enables direct communication between a doctor and patient who are not physically in the same place, typically via videoconferencing tools. However, the patient still interacts with a *human* doctor.

Moreover, artificial intelligence (AI) systems have been discussed, as well as implemented, in various medical contexts such as automatic disease diagnosis or surgeries by robots (e.g., Hashimoto et al., 2018; Kelly et al., 2019). In line with Rai et al. (2019), we define AI “as the ability of a machine to perform cognitive functions that we associate with human minds, such as perceiving, reasoning, learning, interacting with the environment, problem solving, decision-making, and even demonstrating creativity” (p. iii). Importantly, the use of AI systems also spans situations in which a patient interacts with a doctor. In addition to the traditional face-to-face doctor-patient interaction, two AI-based interaction styles have been discussed in the scientific literature and could constitute possible interaction scenarios in the future. First, an AI system *complements* the doctor’s decision regarding diagnosis and/or treatment. Here, the AI system makes a recommendation to the doctor, who verifies the suggestion, overrules it if deemed necessary, and makes the final decision (e.g., Jussupow et al., 2021). Thus, such an AI system can be considered as a decision support system which is used when a doctor interacts with a patient. Second, an AI system *substitutes* the doctor. Here, the patient interacts with an “intelligent” system, but not with a human doctor (e.g., Yokoi et al., 2021). Interaction with such an “intelligent” system typically manifests as patient-chatbot interaction. In addition to computing power and an AI component, such a chatbot has a user interface, which is either text-based (i.e., the patient types on a keyboard and the AI system responds via the screen; comparable to ChatGPT) or speech-based (spoken communication instead of written, comparable to speech assistants like Amazon’s Alexa or Apple’s Siri) (e.g., Powell, 2019). Thus, what we can observe today are different degrees of AI involvement in doctor-patient interaction, with no involvement in classic human-human interaction, some involvement in situations where the doctor is assisted by the AI system, and maximum involvement in situations where the patient interacts only with a machine (i.e., chatbot).

In this article, we report on an online experiment in which participants were asked to imagine they had been suffering from medical conditions of unknown origin for some time and that they were therefore visiting a health center to seek advice from a doctor. We manipulated the patient’s interaction partner—it was either a (i) human doctor, (ii) human doctor with AI system, or (iii) AI system only (i.e., a chatbot). Moreover, we also considered different medical disciplines: (i) cardiology, (ii) orthopedics, (iii) dermatology, and (iv) psychiatry to identify a possible interaction effect of the patient’s interaction partner by medical discipline. The reason for this nuanced perspective is that medical disciplines function differently in terms of the nature of doctor-patient interaction. Psychiatric care, in particular, heavily relies on direct, empathetic human interactions, as these interactions are crucial for the diagnosis and treatment of psychiatric disorders, which are often stigmatized and require nuanced understanding and empathy from the caregiver (e.g., Starke et al., 2021, 2023). Patients in psychiatric settings may perceive a doctor’s use of AI as a distraction, potentially reducing their sense of being cared for and understood, which are vital components of effective psychiatric treatment. In contrast, other medical disciplines such as cardiology, orthopedics, and dermatology involve conditions more directly related to body structures and hence may be less dependent on the emotional and empathetic exchanges critical in psychiatry. AI systems in these fields can assist in diagnosing and treating conditions where objective data and clear physiological markers are paramount, possibly improving efficiency without significantly impacting patient trust, satisfaction, or other important factors in doctor-patient interaction (e.g., Powell, 2019; Nagy and Sisk, 2020). Furthermore, AI’s limitations in interpreting the complex and often subjective nature of psychiatric symptoms highlight the need for cautious integration of AI in psychiatric care. The inability of AI to fully grasp the intricacies of human emotions and social cues can lead to patient distrust and dissatisfaction, especially when privacy concerns and the potential for data breaches are at play (e.g., Asan et al., 2020). Given these differences, it is imperative to consider the specific requirements and patient perceptions in various medical disciplines when integrating AI into doctor-patient interaction.

Against this background, in the present study the participants were presented with 12 (3 × 4) lifelike descriptions of patient-doctor interactions and were asked to respond to questions after being confronted with each situation. The questions referred to six variables: trust, distrust, privacy invasion, information disclosure, treatment adherence, and satisfaction. We chose these six variables as they constitute major factors in patient-doctor interaction (Pearson and Raeke, 2000; Dalton-Brown, 2020; Nagy and Sisk, 2020; Wu et al., 2022).

One seminal conceptualization of trust defines interpersonal trust as the willingness of one party (i.e., the trustor) to be vulnerable to another (i.e., the trustee) based on the belief that the trustee will perform actions that are important to the trustor (Mayer et al., 1995). It follows that the patient is the trustor and the doctor is the trustee in the context of physician-patient interaction. More specifically, in this relationship trust includes the patient’s confidence in the physician’s competence, integrity, and commitment to act in the patient’s best interest (e.g., Birkhäuer et al., 2017). Moreover, it includes the belief that the physician is knowledgeable, skilled, and able to provide effective care while being honest and respectful (e.g., Pearson and Raeke, 2000). Trust is also built through the physician’s ability to listen to and address the patient’s concerns and preferences and especially through confidentiality; therefore, trust enables patients to feel comfortable sharing personal information and following medical advice, ultimately contributing to better health outcomes and patient satisfaction (Pearson and Raeke, 2000; Birkhäuer et al., 2017; Powell, 2019; Gille et al., 2020). Notably, while the importance of trust has been recognized by scholars studying the doctor-patient relationship, the importance of distrust has not (e.g., Birkhäuer et al., 2017). A main reason for this lack of research is that scholars often consider trust and distrust as the opposite ends of a single construct continuum (e.g., Barber, 1983). In contrast, more recent behavioral research, both theoretical (e.g., Lewicki et al., 1998) and empirical (e.g., McKnight and Choudhury, 2006), identifies trust and distrust as separate constructs. Brain research has even found that trust and distrust perceptions activate different brain areas, which is considered the most direct evidence for the fact that trust and distrust are indeed separate constructs, despite the fact that self-report data on trust and distrust may be negatively correlated (Dimoka, 2010; Riedl and Javor, 2012; Krueger and Meyer-Lindenberg, 2019).

Moreover, to control for possible confounding effects, we measured some health status variables, demographics (e.g., sex, age), and several individual difference variables (e.g., personality, technology attitude) of our participants. Controlling for these variables is important because trust and distrust, as well as the other variables which are important in the doctor-patient interaction (i.e., privacy invasion, information disclosure, treatment adherence, and satisfaction), have been shown to correlate with sex (e.g., Riedl et al., 2010), age (e.g., Sutter and Kocher, 2007), personality (e.g., Bruce et al., 2010), and technology attitude (e.g., Nam, 2019), among others.

To the best of our knowledge, no published research has investigated individuals’ preference structure^1^ regarding the 12 manipulations (3 interaction partners × 4 medical disciplines) based on the variables trust, distrust, privacy invasion, information disclosure, treatment adherence, and satisfaction. Therefore, we investigated the following research question: *Do patients prefer a human doctor, artificial intelligence, or a blend, and is this preference dependent on medical discipline?*

The present study contributes to the academic literature in a novel way, and—considering the sharp increase of AI systems in medical contexts in the recent past—also deals with a topic of significant practical relevance. The present study’s contribution is further substantiated by conflicting perspectives and findings in the academic literature. On the one hand, the literature provides sound arguments why patients could prefer AI systems to human doctors. As an example, Dalton-Brown (2020) writes: “A patient may trust an AI more, believing the AI’s health advice is unbiased and not prone to human fallibility. An AI doctor will not be exhausted after a long shift, nor has it any concept of power” (p. 118). On the other hand, recent evidence suggests that an AI system is trusted less than a human doctor in medical treatment decisions (Yokoi et al., 2021). In addition to these conflicting positions regarding patient preferences for a human doctor versus an AI system, we are unaware of empirical evidence on the effects of a blend of both (i.e., a doctor with an AI system). This research deficit is remarkable, as the literature on AI-assisted decision-making distinguishes direct from indirect user interaction with an AI system. In a recent review paper, Vereschak et al. (2021) report that 94% of the extant literature on AI-assisted decision-making examined direct interaction. Thus, also studying indirect interaction is an important research endeavor. Specifically, a patient does not interact directly with the AI system, but with a doctor who is supported by an AI system, and hence from the patient’s perspective the interaction with the system is indirect.

## 2 Materials and methods

### 2.1 Research approach, data collection, and sample

This study conforms to the Declaration of Helsinki.^2^ All participants gave informed consent. We conducted an online experiment with vignettes based on a within-subjects design. Vignettes are “stories about individuals and situations which make reference to important points in the study of perceptions, beliefs, and attitudes” (Hughes, 1998, p. 381). Thus, vignettes are “simulations of real events” (Robert et al., 2009, p. 251). Vignettes have been applied successfully to study medical phenomena (e.g., Gould, 1996; Hughes, 1998).

In the current study, participants were presented 12 different vignettes: 3 patient’s interaction partner (human doctor, human doctor with AI system, AI system alone) × 4 medical discipline (cardiology, orthopedics, dermatology, psychiatry) scenarios in which a patient-doctor interaction was described.^3^ The vignettes are shown in Appendix A (note that all appendices to this paper are part of the Supplementary material). When developing the vignettes, care was taken to ensure that they differed only in the two manipulated variables (patient’s interaction partner, medical discipline). The rest of the text was identical except for linguistic nuances (especially the description of symptoms was kept constant in each medical discipline across the three interaction partner conditions). After the first draft of the vignettes had been developed by the first author, these drafts were checked and revised with the other two co-authors for content and linguistic precision. In addition, the final set of vignettes developed by the authors was also discussed with five practitioners, which led to minor linguistic adjustments (e.g., the term “tachycardia” in the vignettes was replaced by the commonly understood description “a racing heart”). Participants were asked to put themselves in the patient role when answering the questions on trust, distrust, perceived privacy invasion, information disclosure, treatment adherence, and satisfaction. These questions were presented directly after each vignette. Thus, participants responded to the questions regarding these six constructs twelve times.

Moreover, we collected data on the health status^4^ and demographics (sex, age, educational attainment) before the presentation of the vignettes. The vignettes, as well as the questions on all latent constructs, were presented in randomized order. The latent constructs of the present study are: trust, distrust, privacy invasion, information disclosure, treatment adherence, satisfaction, as well as the individual difference variables: personality, disposition to trust humans, disposition to trust machines, technology attitude, and AI phobia. On the first page of the online questionnaire we provided a definition for the term “chatbot” (which was used in several item formulations) to secure a common understanding among the study participants and we also explained the study context.^5^

Data were collected in 2022 by a market research company.^6^ Respondi, originally a German company, was recently acquired by the French market research company Bilendi,^7^ but both brands continue to exist. Respondi provides services such as online surveys and access to diverse panels to support both academic research and market research. The company is ISO quality certified (ISO 20252:2019). The target population of the online experiment were individuals from the German-speaking area (Austria, Germany, Switzerland) with a minimum age of 18 years. All statistical analyses presented in the current paper are based on a total sample of *N* = 1,183 persons.^8^ The median participation time in our online experiment was 29 min. Table 1 provides an overview of the main sample characteristics.

**Table 1:** Overview of main sample characteristics (*N* = 1,183).

Country (current residence)	Austria: 386 (33%), Germany: 441 (37%), Switzerland: 351 (30%), other: 1 (<1%)
Sex	Female: 648 (55%), male: 535 (45%)
Age (years)	18–19: 244 (21%), 30–39: 220 (19%), 40–49: 213 (19%), 50–59: 263 (23%), >60: 203 (18%)^1^
Educational attainment	No degree (6, <1%), compulsory education (47, 4%), apprenticeship / professional training (497, 42%), high school diploma (309, 26%), university degree (324, 28%)
Self-reported Health status	Generally chronically ill [yes: 711 (60%), no: 472 (40%)], mentally ill [yes: 135 (11%), no: 1,048 (89%)], orthopedically ill [yes: 135 (11%), no: 1,048 (89%)], dermatologically ill [yes: 70 (6%), no: 1,113 (94%)], illness in the domain of internal medicine [yes: 161 (14%), no: 1,022 (86%)], other illness [yes: 134 (11%), no: 1049 (89%)] (multiple answers possible)
Regular intake of medication^2^	Yes: 469 (40%), no: 713 (60%)

^1^Due to missing, incorrect or ambiguous information on age in the data set, only a sample of *N* = 1,143 was used for the description of the age groups as well as for analyses that include age.

^2^This also includes contraceptive.

### 2.2 Measures

All latent constructs were measured based on psychometrically validated instruments which also showed high reliability in the present sample (Cronbach’s Alpha > 0.7). Specifically, we used the following instruments with some wording adjustments to exactly fit the present research context: trust (Söllner et al., 2012) (5 items, sample item: “I have a good feeling when relying on the diagnosis decision.”), distrust (Söllner et al., 2012) (5 items, sample item: “I have a bad feeling when relying on the treatment decision.”), privacy invasion (Fischer et al., 2021) (4 items, sample item: “I fear that my interaction with the [physician/physician who is supported by the chatbot/chatbot] is less confidential than I would like it to be.”), information disclosure (Bansal et al., 2010) (3 items, sample item: “I have a strong intention to disclose health information to the [physician/physician who is supported by the chatbot/chatbot].”), treatment adherence (DiMatteo et al., 1992) (4 items, sample item: “I will follow the [physician’s/chatbot supported physician’s/chatbot’s] suggestions exactly.”), satisfaction (Probst et al., 1997) (4 items, sample item: “In general, I would be satisfied with this type of diagnosis and treatment development method.”), personality (Gosling et al., 2003) (big-five model, two items for each factor = 10 items, sample item for neuroticism: “I easily get nervous and insecure.”), disposition to trust humans (Gefen, 2000) (4 items, sample item: “I generally have faith in humanity.”), disposition to trust machines (Gefen, 2000) (4 items, sample item, “I tend to count upon machines.”), technology attitude (Nam, 2019) (2 items, sample item, “Thinking about the possibility that computers and robots could do most of the work currently done by humans, how enthusiastic are you, if at all, about this possibility for society as a whole? not at all enthusiastic / not too enthusiastic / somewhat enthusiastic / very enthusiastic.”), and AI phobia (Khasawneh, 2018) [7 items, sample item: “I am afraid of new technologies because one day they will make us (humans) obsolete.”]. Appendix B summarizes the measurement instruments, including all items and Cronbach’s Alphas.^9^ Appendix C shows the correlation table with the six main factors investigated.

### 2.3 Data analyses

**Figure 1:**
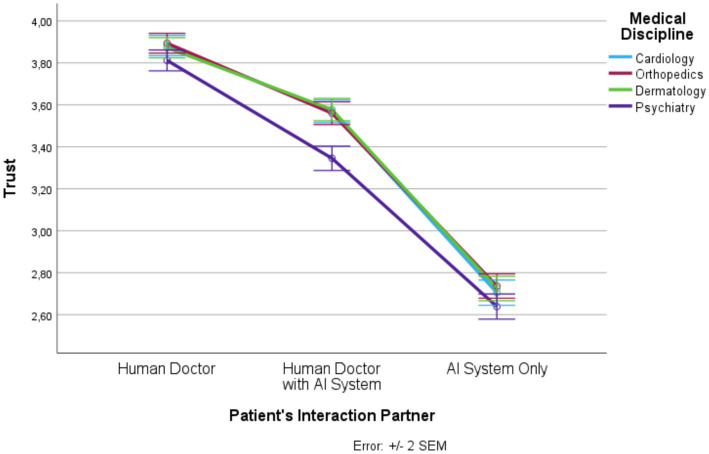
Patient’s interaction partner and trust.

**Figure 2:**
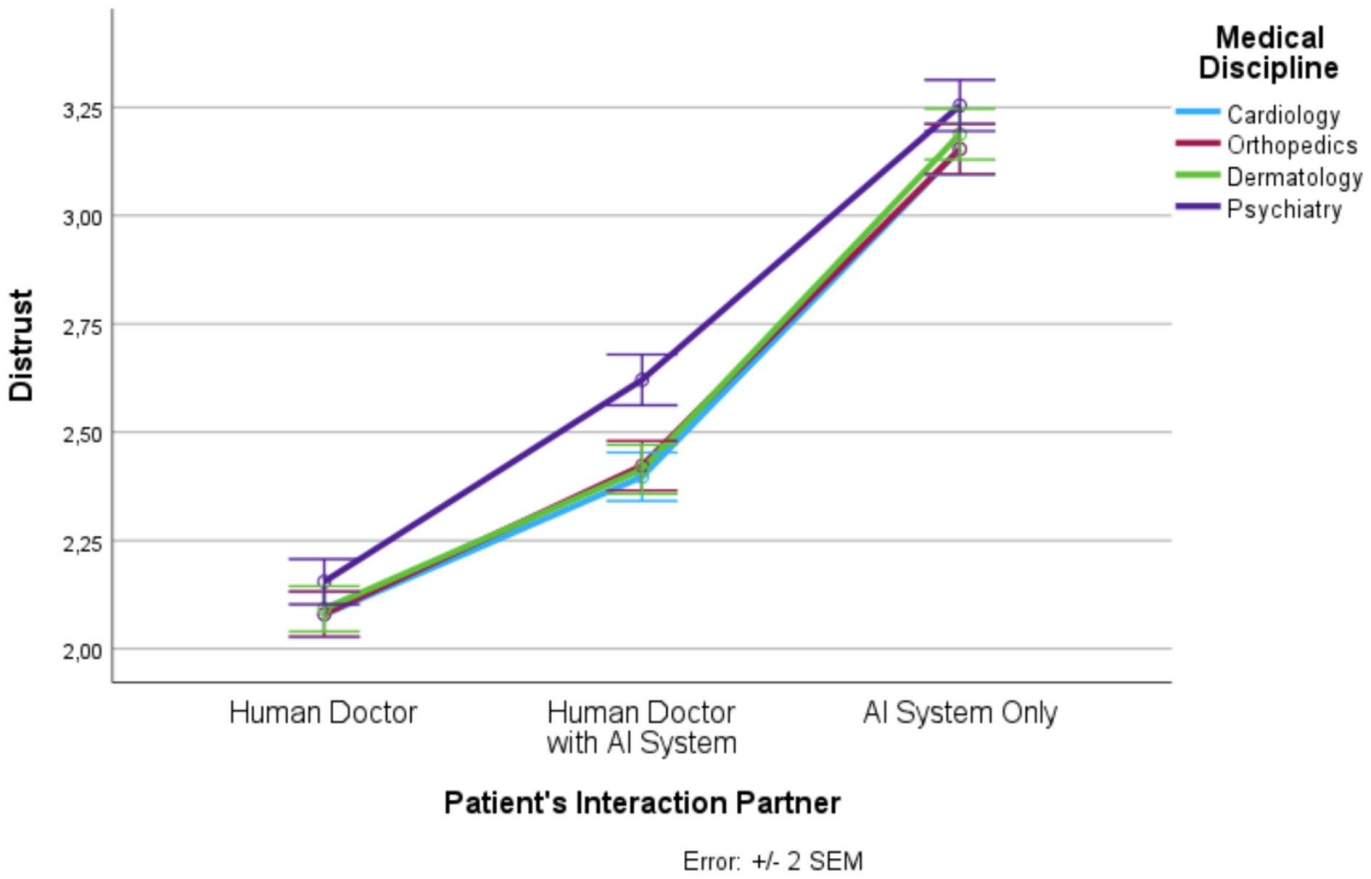
Patient’s interaction partner and distrust.

**Figure 3:**
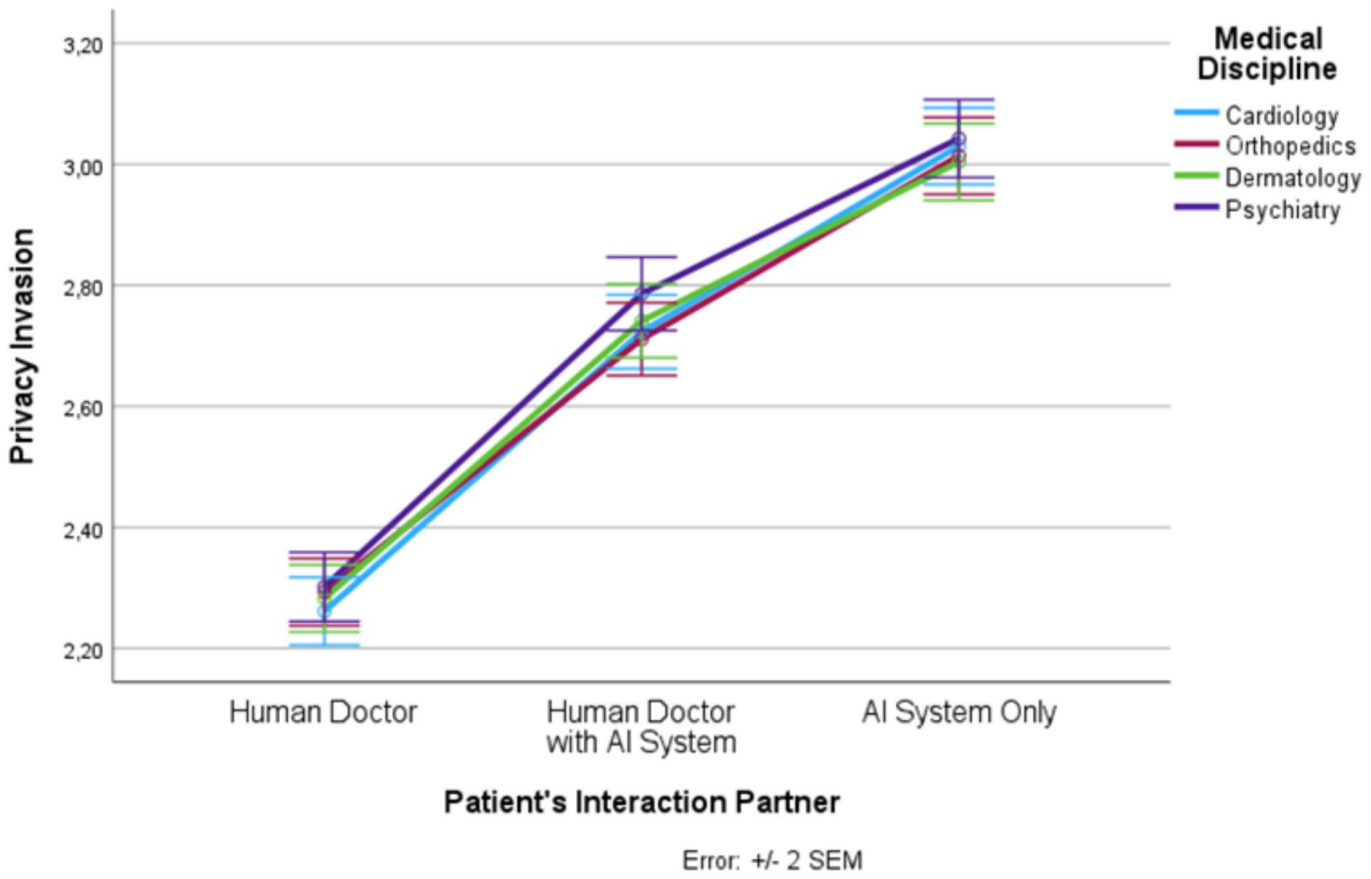
Patient’s interaction partner and privacy invasion.

**Figure 4:**
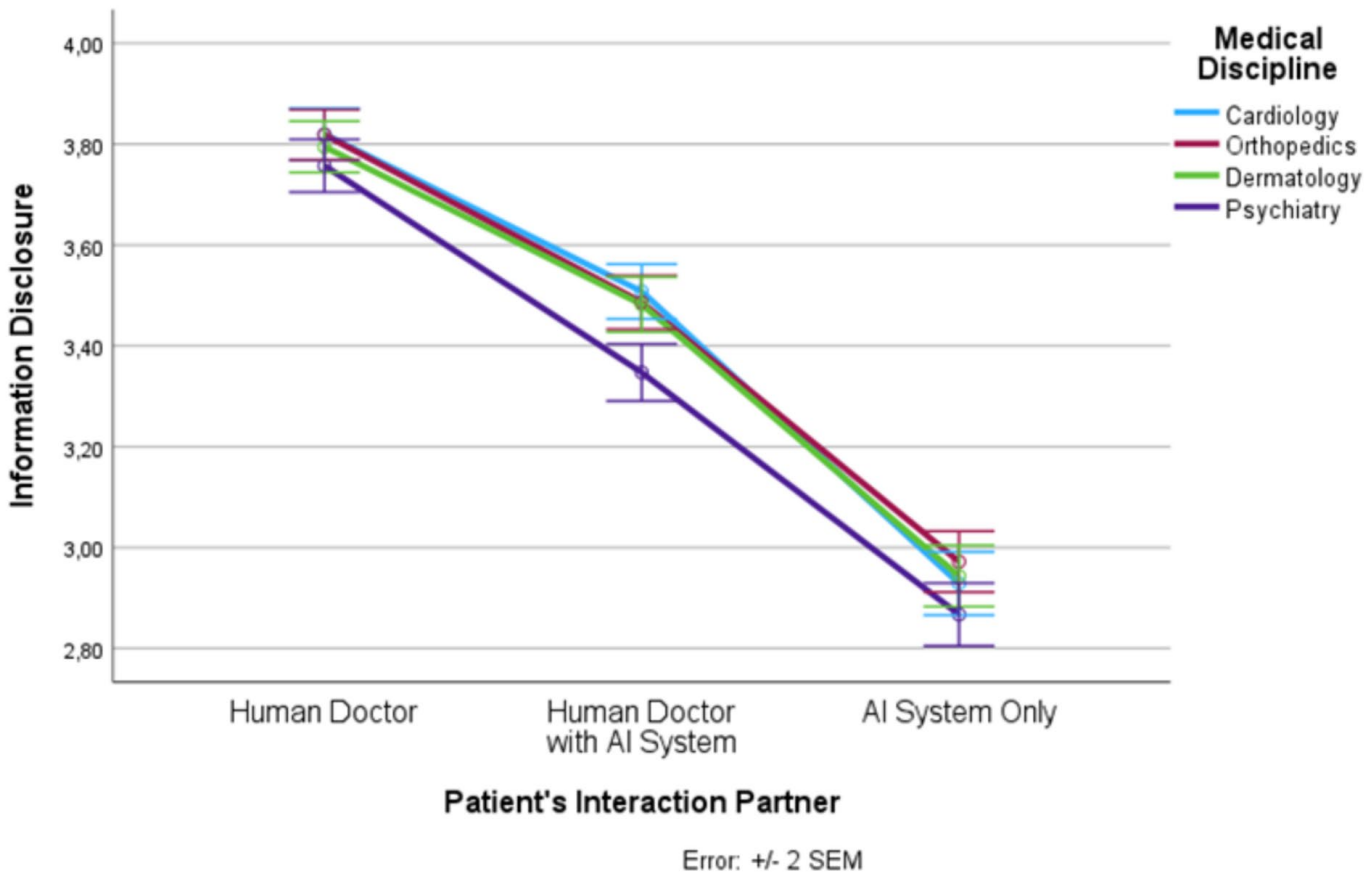
Patient’s interaction partner and information disclosure.

**Figure 5:**
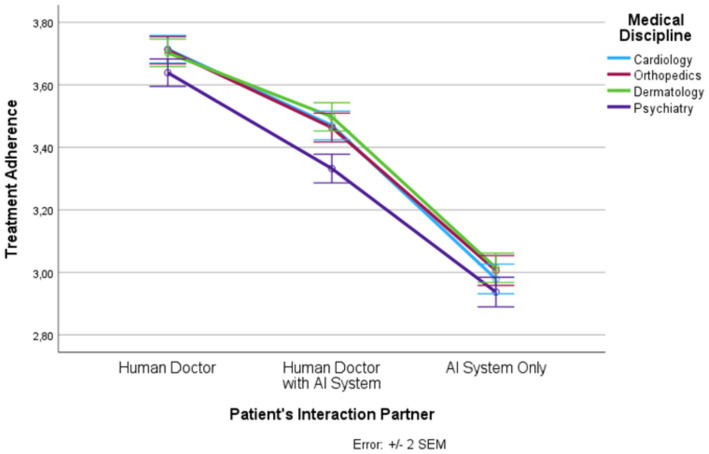
Patient’s interaction partner and treatment adherence.

**Figure 6:**
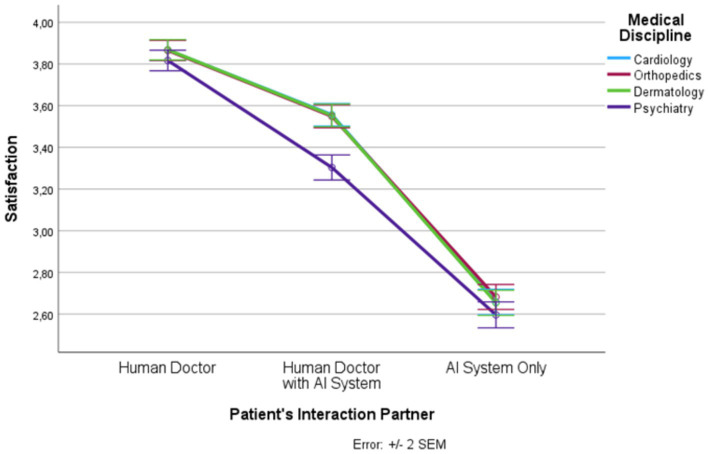
Patient’s interaction partner and satisfaction.

Based on SPSS 27 repeated measures analysis of variance (RM-ANOVA) was conducted to determine the influence of the two within-subject factors, patient’s interaction partner (human doctor, human doctor with AI system, AI system only) and medical discipline (cardiology, orthopedics, dermatology, psychiatry) on trust, distrust, privacy invasion, information disclosure, treatment adherence, and satisfaction. For each dependent variable a separate RM-ANOVA was calculated. The description of the mean values and standard errors of the mean (SEM) values are visualized graphically in Figures 1–6 for each dependent variable and summarized in Appendix D. Tests of the within-subject effects and interaction effects are reported. To address violations of the sphericity assumption, the Greenhouse–Geisser correction to adjust the degrees of freedom was applied if the result of the Mauchly test reaches significance. Bonferroni tests were used as *post hoc* tests to correct for multiple comparisons. In addition to these RM-ANOVA models we calculated additional analyses of covariance (ANCOVAs) controlling for possible confounding effects (see Appendix E). Moreover, as outlined in Appendix F, based on LISREL v. 8.80 structural equation modeling (SEM) was applied. SEM was used to model and test that perceptions of trust, distrust, and privacy invasion predict information disclosure, treatment adherence, and satisfaction. The statistical foundations of our approach can be found in Jöreskog (1970); further details and statistical results are presented in Appendix F.

## 3 Results

### 3.1 Analysis of variance (RM-ANOVA)

In the following, we outline the RM-ANOVA results. The presentation is organized along the six factors, first showing the results for the main effect of the interaction partner, then the effects of the medical discipline, and finally we report the interaction effects between the two factors. Moreover, Figures 1–6 and Appendix D show the mean and SEM values for each factor.

#### 3.1.1 Trust

Results show a strong main effect of the patient’s interaction partner on trust (*F* (1.55, 1835.06) = 943.63, *p* ≤ 0.0001, η_p_^2^ = 0.444; see Figure 1). In total, more than 44% of the variance in trust could be explained by the patient’s interaction partner. We observed highest trust in the “human doctor” condition, lowest trust in the “AI system only” condition, and intermediate trust in the “human doctor with AI system” condition (*F* (1, 1,182) = 1179.57, *p* ≤ 0.0001, *η*^2^ = 0.499). All three levels differed significantly from each other.

Medical discipline explained about 5% of the variance in trust (*F* (2.77, 3277.31) = 64.64, *p* ≤ 0.0001, η_p_^2^ = 0.052). Psychiatry had significantly lower trust scores than the other three disciplines. In addition, there was an interaction effect (*F* (5.76, 6812.50) = 11.10, *p* ≤ 0.0001, η_p_^2^ = 0.009). For all levels of the patient’s interaction partner, the trust scores were significantly lower in psychiatry than in the other three medical disciplines but this effect was strongest in the “human doctor with AI system” condition.

#### 3.1.2 Distrust

Results show a strong main effect of the patient’s interaction partner on distrust (*F* (1.55, 1830.39) = 799.16, *p* ≤ 0.0001, η_p_^2^ = 0.403; see Figure 2). In total, 40% of the variance in distrust could be explained by the patient’s interaction partner. We observed lowest distrust in the “human doctor” condition, highest distrust in the “AI system only” condition, and intermediate distrust in the “human doctor with AI system” condition (*F* (1, 1,182) = 1012.41, *p* ≤ 0.0001, η_p_^2^ = 0.461). All three levels differed significantly from each other.

Medical discipline explained about 4% of the variance in distrust (*F* (2.84, 3353.71) = 53.94, *p* ≤ 0.0001, η_p_^2^ = 0.044). Psychiatry had significantly higher distrust scores than the other three disciplines. In addition, there was an interaction effect (*F* (5.77, 6820.03) = 7.65, *p* ≤ 0.0001, η_p_^2^ = 0.006). For all levels of the patient’s interaction partner, the distrust scores were significantly higher in psychiatry than in the other three medical disciplines but this effect was strongest in the “human doctor with AI system” condition.

#### 3.1.3 Privacy invasion

Results show a strong main effect of the patient’s interaction partner on privacy invasion (*F* (1.41, 1660.44) = 483.05, *p* ≤ 0.0001, η_p_^2^ = 0.290; see Figure 3). In total, 29% of the variance in privacy invasion could be explained by the patient’s interaction partner. We observed lowest privacy invasion in the “human doctor” condition, highest privacy invasion in the “AI system only” condition, and intermediate privacy invasion in the “human doctor with AI system” condition (*F* (1, 1,182) = 599.02, *p* ≤ 0.0001, η_p_^2^ = 0.336). All three levels differed significantly from each other.

Medical discipline explained 0.5% of the variance in privacy invasion (*F* (3, 3,546) = 6.32, *p* ≤ 0.001, η_p_^2^ = 0.005). Psychiatry had significantly higher privacy invasion scores than the other three disciplines. In addition, there was an interaction effect (*F* (5.93, 7010.83) = 2.12, *p* = 0.049, η_p_^2^ = 0.002). However, this interaction effect was only marginal. Privacy invasion was significantly higher for the medical discipline psychiatry compared with cardiology and orthopedics (but differed not significantly from dermatology), but this effect only occurred in the “human doctor with AI system” condition.

#### 3.1.4 Information disclosure

Results show a strong main effect of the patient’s interaction partner on information disclosure (*F* (1.46, 1725.46) = 590.69, *p* ≤ 0.0001, η_p_^2^ = 0.333; see Figure 4). In total, more than 33% of the variance in information disclosure could be explained by the patient’s interaction partner. We observed highest information disclosure in the “human doctor” condition, lowest information disclosure in the “AI system only” condition, and intermediate information disclosure in the “human doctor with AI system” condition (*F* (1, 1,182) = 726.64, *p* ≤ 0.0001, η_p_^2^ = 0.381). All three levels differed significantly from each other.

Medical discipline explained about 3% of the variance in information disclosure (*F* (2.88, 3398.21) = 34.55, *p* ≤ 0.0001, η_p_^2^ = 0.028). Psychiatry had significantly lower information disclosure scores than the other three disciplines. In addition, there was an interaction effect (*F* (5.85, 6909.81) = 4.16, *p* ≤ 0.001, η_p_^2^ = 0.004). For all levels of the patient’s interaction partner, the information disclosure scores were significantly lower in psychiatry than in the other three medical disciplines (except for the pairwise comparison between dermatology and psychiatry in the “human doctor” condition) but this effect was strongest in the “human doctor with AI system” condition.

#### 3.1.5 Treatment adherence

Results show a strong main effect of the patient’s interaction partner on treatment adherence (*F* (1.59, 1873.14) = 590.68, *p* ≤ 0.0001, η_p_^2^ = 0.333; see Figure 5). In total, more than 33% of the variance in treatment adherence could be explained by the patient’s interaction partner. We observed highest treatment adherence in the “human doctor” condition, lowest treatment adherence in the “AI system only” condition, and intermediate treatment adherence in the “human doctor with AI system” condition (*F* (1, 1,182) = 764.02, *p* ≤ 0.0001, η_p_^2^ = 0.393). All three levels differed significantly from each other.

Medical discipline explained about 3% of the variance in treatment adherence (*F* (2.89, 3421.20) = 37.82, *p* ≤ 0.0001, η_p_^2^ = 0.031). Psychiatry had significantly lower treatment adherence scores than the other three disciplines. In addition, there was an interaction effect (*F* (5.95, 7026.41) = 4.18, *p* ≤ 0.001, η_p_^2^ = 0.004). For all levels of the patient’s interaction partner, the treatment adherence scores were significantly lower in psychiatry than in the other three medical disciplines (except for the pairwise comparison between cardiology and psychiatry in the “AI system only” condition) but this effect was strongest in the “human doctor with AI system” condition.

#### 3.1.6 Satisfaction

Results show a strong main effect of the patient’s interaction partner on satisfaction (*F* (1.56, 1844.44) = 865.17, *p* ≤ 0.0001, η_p_^2^ = 0.423; see Figure 6). In total, more than 42% of the variance in satisfaction could be explained by the patient’s interaction partner. We observed highest satisfaction in the “human doctor” condition, lowest satisfaction in the “AI system only” condition, and intermediate satisfaction in the “human doctor with AI system” condition (*F* (1, 1,182) = 1085.348, *p* ≤ 0.0001, η_p_^2^ = 0.479). All three levels differed significantly from each other.

Medical discipline explained 4.9% of the variance in satisfaction (*F* (2.76, 3263.35) = 60.85, *p* ≤ 0.0001, η_p_^2^ = 0.049). Psychiatry had significantly lower satisfaction scores than the other three disciplines. In addition, there was an interaction effect (*F* (5.73, 6777.95) = 17.57, *p* ≤ 0.0001, η_p_^2^ = 0.015). For all levels of the patient’s interaction partner, the satisfaction scores were significantly lower in psychiatry than in the other three medical disciplines but this effect was strongest in the “human doctor with AI system” condition.

### 3.2 Structural equation model with latent variables

Evidence suggests that trust, along with distrust and privacy invasion, may serve as predictors of information disclosure, treatment adherence, and satisfaction [see, for example, the evidence reported in a meta-analysis by Birkhäuer et al. (2017)]. Given the theoretical background of the present study, we investigated the mentioned prediction as a function of the patient’s interaction partner and medical discipline. To do this, we built a structural equation model (SEM). We calculated a mixed model, i.e., we combined measurement models of the dependent variables and predictors with the assessment of the relationship between their latent factors. Table 2 summarizes the standardized regression coefficients (𝛽), standard errors of regression (SER), and the t-values.

**Table 2:** Total effects of predictors on dependent variables.

			Predictors		
			Trust	Distrust	Privacy invasion
Dependent variables	Information disclosure	𝛽	1.03	0.36	−0.31
		SER	0.11	0.11	0.06
		*t*-value	9.23	3.36	−4.77
	Treatment adherence	𝛽	0.45	−0.56	−0.04
		SER	0.08	0.12	0.06
		*t*-value	5.49	−4.72	−0.63
	Satisfaction	𝛽	1.10	—	—
		SER	0.08	—	—
		*t*-value	13.96	—	—

The “—” sign in the table indicates that neither distrust nor privacy invasion has a statistically significant influence on satisfaction. Standardized regression coefficients: 𝛽. Standard errors of regression: SER.

Moreover, for *R*^2^ we found the following results: information disclosure 0.99, treatment adherence 0.95, and satisfaction 0.24. What follows is that trust, distrust, and privacy invasion *together* explain significant variance in the three dependent variables; in case of information disclosure 99% and in case of treatment adherence 95%, a remarkable result, and in case of satisfaction they still explain approximately a quarter of the total variance (24%).

In addition to Table 2, we present further statistical results in Appendix F based on SEM and LISREL modeling.

## 4 Discussion

In the current study, we first investigated the question whether patients prefer a human doctor, a human doctor with an AI system, or an AI system alone. Thus, our study contributes to a better understanding of the acceptance of possible new forms of patient-doctor interaction and the consequences of a possible future shift from classical human-human interaction to interaction scenarios in which AI systems play a role, either as decision support for the doctor or as a completely autonomous chatbot (i.e., machine). We found that people prefer a human doctor, followed by a human doctor with an AI system, and an AI system alone came in last place. Specifically, trust, information disclosure, treatment adherence, and satisfaction were significantly higher and distrust and perceived privacy invasion were significantly lower in the “human doctor” condition than in the “human doctor with AI system” and “AI system alone” conditions. We observed the same result pattern when comparing the “human doctor with AI system” and the “AI system alone” conditions.

The implications of these findings for medical practice are far-reaching. Our results strongly suggest that patients prefer to interact with a human doctor *without* AI support. Therefore, while the enormous potential of AI systems in medical contexts such as automatic disease diagnosis or surgeries by robots is undeniable (e.g., Hashimoto et al., 2018; Kelly et al., 2019), interaction with a doctor who uses an AI system during the conversation with the patient, and even more direct interaction with an AI system without a human doctor at all, likely entails negative consequences and reduces the potential therapy success. Moreover, our results suggest that the negative attitude toward AI-supported, or AI-based, interaction in patient-doctor communication processes could be mediated by psychological variables related to trust (see Appendix F).

Our results also suggest that patients, if eventually enforced to interact with “intelligent” machines in the future (e.g., due to cost pressure in the health care sector), will likely engage in behaviors which could endanger their health, such as reduced information disclosure in anamnesis or reduced treatment adherence. Thus, substitution of human doctors by AI systems could ultimately result in higher costs (e.g., due to later diagnosis of diseases or because medication cannot be effective due to lack of treatment adherence). Moreover, based on our results, it is also likely that patient satisfaction will suffer when they have to interact with AI systems directly, or with a doctor who is supported by an AI system. Altogether, our data suggest that patients prefer direct face-to-face communication in their interaction with doctors.

Recent evidence indicates that human interaction partners are preferred to AI systems for different reasons: the ability to feel emotions (Bigman and Gray, 2018), to avoid perceptions of uniqueness neglect (Longoni et al., 2019), perceived care and value similarity (Yokoi et al., 2021), and the ability to demonstrate empathy and to be benevolent (Wu et al., 2022). Consistent with these reasons, research on classical human-computer interaction situations (e.g., user interacts with a desktop PC), as well as research in non-medical AI contexts such as autonomous vehicle use, found that “humanizing technology” (e.g., by simply giving the machine a human name or by endowing a machine with complex behaviors such as demonstrating empathy or reciprocity) may significantly increase trust in the technology, which typically positively affects acceptance and adoption of the technology (e.g., Brave et al., 2005; Waytz et al., 2014). Considering these findings, future research on patient interaction with an AI system could experimentally manipulate the machine’s characteristics to resemble typical human attributes. The prediction is that trust in the AI system would increase (and we also expect reduced distrust and privacy invasion perceptions). However, research in this context must not ignore ethical principles. In particular, if empirical research confirms the prediction that “humanizing technology” positively affects its acceptance and adoption, a broad discussion in medicine, as well as in society at large, should follow before designers and engineers create AI-based technologies that influence humans in ethically questionable ways in situations where a human doctor is replaced by a machine.

Furthermore, our study revealed that the scores for the six outcome variables differed significantly between psychiatry and the three other disciplines (cardiology, orthopedics, dermatology). Specifically, we found lower scores for trust, information disclosure, treatment adherence, and satisfaction, and higher scores for distrust and privacy invasion. Notably, we observed an interaction effect of patient’s interaction partner by medical discipline. This interaction effect was caused by the fact that the six outcome variables differed strongly between psychiatry and the three other disciplines in the “human doctor with AI system” condition, while this effect was not that strong in the other conditions (human doctor, AI system alone). This result suggests that the human doctor alone condition is appreciated and the AI system alone is disliked almost equally across all medical disciplines. However, in the hybrid condition (human doctor with AI system) the nature of the disease or disorder makes a difference. The diagnosis and treatment of psychiatric disorders, which are often not discussed openly (because a psychiatric problem is eventually more stigmatized than a physical illness), are based more on the direct interaction between doctor and patient (i.e., human-human interaction) than the diagnosis and treatment of illnesses in other medical disciplines. Also, the pathological thought patterns underlying psychiatric disorders are likely to be less clearly identifiable by AI algorithms, and less amenable to AI algorithmic influence, than diseases more directly related to body structures (e.g., heart, spine, skin). This could explain why patients dislike interaction with a doctor who is supported by an AI system more in psychiatry than in the other investigated disciplines, and this difference does not exist when a patient interacts with a human doctor or with an AI system only.

These results are consistent with the mentioned reasons why people prefer human interaction partners to AI systems. In psychiatry, human characteristics such as the ability to feel emotions, perceived care, or the ability to demonstrate empathy and to be benevolent are comparatively more important than in other medical disciplines because exchange of communicative stimuli and social cues are *the* processes of utmost importance in the interaction between the patient and the doctor, as signified, for example, by doctor-patient interaction in psychotherapy. It seems that people believe that when a psychiatrist uses an AI system as decision support during the interaction process, the doctor’s attention shifts from the patient to the system, possibly reducing patient perceptions such as perceived care, empathy, and benevolence. In essence, patients with psychiatric conditions may prefer to interact with a human doctor for various reasons. The present study suggests that trust and human connection play an important role. Concerns about confidentiality and privacy may also influence preferences, and our data suggest that patients fear data breaches in the doctor with AI system and in the AI system only conditions more than in the human doctor condition.

Altogether, our finding on the interaction effect of a patient’s interaction partner by medical discipline suggests a “stuck-in-the-middle” issue in psychiatry. The practical implication of this issue is that if possible, psychiatrist-patient interaction should take place face-to-face. If this is not possible or necessary, computer-mediated interaction (e.g., via videoconferencing) (e.g., Backhaus et al., 2012) could be chosen. Importantly, recent evidence shows that interaction with a fully automated AI chatbot could be an effective approach in the diagnosis and treatment of psychiatric disorders (e.g., Lee et al., 2022). This finding, however, is not supported by the results of our study because, at least based on the six factors examined, people clearly prefer a human doctor to a non-human chatbot. Future research is necessary to establish the exact reasons for differences in research findings. As a starting point, we recommend recent papers that address the ethics of using AI technology, particularly machine learning, in psychiatric settings (Starke et al., 2021, 2023), as well as related papers on topics such as machine learning in predicting treatment outcomes in psychiatry (Chekroud et al., 2021).

The present study has limitations, several of which could also be addressed in future research. First, our participants did not respond to interaction with a real doctor, a real doctor with AI system, or a real AI system. As a result of our vignette-based research approach, participants could eventually respond differently in the real world, if compared to our hypothetical scenarios. However, Robert et al. (2009) report evidence showing that results of vignette studies and non-vignette studies are similar in decision-making contexts, and based on this evidence they argue that “when individuals are presented with vignettes, they respond as they do in a real scenario [… yet] vignettes can never mirror completely the reality and dynamism of people’s lives” (p. 267). Despite this limitation, we emphasize that the vignette-based approach constitutes an established method in the study of decision-making phenomena in the medical context (Gould, 1996; e.g., Hughes, 1998). Moreover, we emphasize that in the present study we did not examine learning of trust. Therefore, based on existing research on the foundations of trust learning (e.g., King-Casas et al., 2005; Krueger and Meyer-Lindenberg, 2019), future research must not ignore the effects of experiences in the interaction between doctor and patient on trust perceptions and the other variables examined in the present study.

Second, the sample of the present study was recruited in German-speaking countries (Austria, Germany, Switzerland). Because evidence indicates that trust is related to culture (e.g., Thanetsunthorn and Wuthisatian, 2019), a fact which also holds true specifically for trust in AI systems (e.g., Chien et al., 2016), future studies should test the generalizability of our findings to identify possible cultural differences.

Third, we found that most control variables only had a marginal influence on the main effect (see Appendix E). In particular, we highlight that this result is unexpected especially with respect to personality (Oksanen et al., 2020). However, personality can be conceptualized and measured in different ways. We conceptualized personality based on the Big Five and used Gosling et al.’s (2003) seminal short-version measurement instrument (i.e., TIPI = Ten Item Personality Measure). In addition to this measurement of universal personality traits, we measured four *specific* personality traits (disposition to trust humans, disposition to trust machines, technology attitude, AI phobia). However, a recent review paper at the nexus of trust in AI systems and personality analyzed *N* = 58 empirical articles and identified five conceptualizations of universal personality traits (e.g., HEXACO) and 33 specific personality traits (Riedl, 2022). What follows is that future research should use other personality conceptualizations and measurement instruments to replicate our finding that personality, in general, hardly affected the main effects (see our RM-ANOVA findings). In corresponding research efforts, scholars should consider a recent research agenda paper on the role of personality in AI systems and robot use (Matthews et al., 2021).

Fourth, another limitation of our study that warrants discussion is the potential for participants to have inaccurate or unjustified perceptions of AI technologies, which may have influenced their responses. Participants’ expectations of AI and its capabilities are also shaped by external factors such as media portrayals and personal biases, leading to unrealistic expectations or undue skepticism. In our vignettes, while we provided a brief description of chatbots, we did not offer a comprehensive explanation of AI systems in general or Clinical Decision Support Systems (CDSS) and their potential roles in assisting doctors (we deliberately avoided this in the present study; we did not want to add to the already long length with additional explanations and definitions). However, this omission might have contributed to participants’ perceptions and preferences being based on incomplete or incorrect information about AI systems and CDSS. Future research should aim to address this by setting more realistic expectations through more detailed descriptions and educational interventions about AI technologies, thereby allowing for a more informed evaluation of their potential impact on the doctor-patient relationship.

Fifth, we cannot rule out some degree of participant inattention. The study was relatively long, and participants had to provide information on the six outcome variables 12 times during the experiment. Although the order of presentation of the vignettes and latent constructs was randomized, which mitigates this potential problem, potential inattention could still affect the results. Given this limitation, potential future replication studies should include attention controls distributed throughout the study. We emphasize, however, that there is no reason to believe that some degree of participant inattention would systematically affect the main findings of the present study, including the main effect of interaction partner on trust, distrust, perceived privacy invasion, information disclosure, treatment adherence, and satisfaction, as well as the finding that the situation in psychiatry differs from that in cardiology, orthopedics, and dermatology.

Sixth and finally, regarding our manipulation “doctor with AI system” we highlight that the present experiment solely focused on the patient’s perspective. Thus, it was not the goal of our study to also investigate the doctor’s trust in the AI system (along with possible perceptions of distrust and privacy invasion). However, it is obvious that such a research focus is also critical in future research because the doctor’s trust along with corresponding beliefs, attitudes, and behaviors could influence trust, distrust, perceived privacy invasion, information disclosure, treatment adherence, and patient satisfaction. As a starting point for corresponding research, we recommend a paper by Asan et al. (2020) who deal with the physician perspective in the study of trust in AI systems, as well as a paper by Jussupow et al. (2021) who empirically examined physicians’ thought processes while using an AI system.

The most important implication of the current study’s results is that *the replacement of a human doctor by an AI system in the doctor-patient discussion should not be carried out without taking into account the possible consequences*. Our conclusion is in line with statements in the academic literature on an ongoing debate regarding use of autonomous AI systems in medicine. Gille et al. (2020), for example, recently wrote that “[h]owever effective, these systems offer few clues as to how they arrive at their conclusions, hence raise questions of transparency, accountability and responsibility” (p. 1). Similarly, Powell (2019) argued that “many medical decisions require value judgments and the doctor-patient relationship requires empathy and understanding to arrive at a shared decision, often handling large areas of uncertainty and balancing competing risks. Arguably, medicine requires wisdom more than intelligence, artificial or otherwise. Artificial intelligence therefore needs to supplement rather than replace medical professionals, and identifying the complementary positioning of artificial intelligence in medical consultation is a key challenge for the future” (p. 1). It will be rewarding to see what insights future research will reveal.

## Data availability statement

The raw data supporting the conclusions of this article will be made available by the authors, without undue reservation.

## Ethics statement

Ethical review and approval was not required for the study on human participants in accordance with the local legislation and institutional requirements. The studies were conducted in accordance with the local legislation and institutional requirements. The participants provided their written informed consent to participate in this study.

## Author contributions

RR: Conceptualization, Funding acquisition, Methodology, Project administration, Validation, Writing – original draft. SH: Data curation, Formal analysis, Visualization, Writing – original draft. MR: Conceptualization, Data curation, Formal analysis, Methodology, Validation, Visualization, Writing – review & editing.

## Footnotes

^1^A preference refers to a person’s attitude towards a specific stimulus.

^2^In our purely behavioral research study, the key ethical considerations from the Declaration of Helsinki include obtaining informed consent, ensuring beneficence and non-maleficence, maintaining confidentiality, conducting risk–benefit assessments, ensuring scientific validity, and ensuring credible, meaningful results.

^3^These four medical disciplines were selected because they differ significantly. Importantly, in the preparation of the present study the authors considered more disciplines (e.g., neurology). However, based on feedback in the pre-testing phase it turned out that 12 different vignettes are the reasonable maximum regarding effort to complete the study. Note that any other discipline would have increased the number of vignettes by an additional 3.

^4^Regarding health status we measured, based on “yes” and “no” answers, the occurrence of illnesses in different domains such as orthopedics, dermatology, or internal medicine. Moreover, we measured whether a participant took medication on a regular basis.

^5^Specifically, we explained that communication with a chatbot alone means to communicate with an automatic computer system (i.e., a machine).

^6^ https://www.respondi.com/

^7^ https://www.bilendi.de/

^8^The data set was complemented by a small sample (*n* = 97) recruited by the authors via online advertisement. These 97 individuals are part of the original sample of *N* = 1,188. However, five persons were excluded because they did not specify some demographic data. Moreover, on the last page of the online questionnaire we asked the following question: “Did you answer the questionnaire carefully and conscientiously?” All 1,183 persons confirmed this question with “yes.”

^9^Note that calculation of reliability coefficients was not possible for personality and technology attitude because each scale only includes two items. Hence, we report intercorrelations for the items of each scale (see Appendix B).
