# Supplementary material for: Do patients prefer a human doctor, artificial intelligence, or a blend, and is this preference dependent on medical discipline? Empirical evidence and implications for medical practice
Source: Front Psychol. 2024 Aug 12;15:1422177. doi: 10.3389/fpsyg.2024.1422177 (PMC11345249; doi:10.3389/fpsyg.2024.1422177)
Supplement: Supplementary file 1 [file Table_1.pdf]

Supplementary Material

Do Patients Prefer a Human Doctor, Artificial Intelligence, or a Blend, and Is This Preference Dependent on Medical Discipline? Empirical Evidence and Implications for Medical Practice

René Riedl, Svea Hogeterp, Martin Reuter

Frontiers in Psychology, section Health Psychology (2024)

This *Supplementary Material* includes:

- Appendix A
- Appendix B
- Appendix C
- Appendix D
- Appendix E
- Appendix F

All references cited in this Supplementary Material are listed in the references of the main paper.

Appendix A

This appendix shows the introductory text and the twelve vignettes as used in the online experiment. The introductory text and the vignettes are also available in the original German language on request.

Introductory text

What will medical care look like in the future?

Digital technologies such as artificial intelligence (AI) and chatbots (i.e., computer systems that communicate with people automatically) are on the rise and are already being used in many areas of life (e.g., online shopping). Digital technologies are also becoming increasingly important in medicine. In this study, we want to investigate what attitudes and opinions people have about possible doctor-patient relationships in the future, and we are particularly interested in different degrees of digitalization in the doctor-patient relationship. Will we communicate directly with a doctor, will the doctor rely on computer-based decisions based on AI, or will we perhaps communicate exclusively with chatbots in the future?

This research project is a cooperative study between the Department of Psychology (Differential & Biological Psychology) at the University of Bonn, Germany, and the Austrian universities University of Applied Sciences Upper Austria, Campus Steyr, and the University Linz.

The survey takes about 30 minutes. Your participation is voluntary and anonymous and can be canceled at any time without giving reasons and without negative consequences. You must be at least 18 years old to participate.

By clicking on "Continue" you agree to participate in the survey and start the questionnaire.

Vignettes

Before the vignettes began, the following introductory text was presented:

We would now like to invite you to take part in a thought experiment: Please imagine that you are in the situations presented on the following pages. Below you will see statements on which you can express your opinion. You will go through a total of twelve different scenarios.

All twelve vignettes were started with the following introduction, followed by the text as shown below. After each vignette, participants were asked to answer the questions on trust, distrust, privacy invasion, information disclosure, treatment adherence, and satisfaction. The vignettes, as well as the questions on all latent constructs, were presented in randomized order.

A new health center has recently opened in the region where you live. Specialists from different disciplines as well as psychotherapists work in this health center. You have come to the health center because you have been suffering from medical conditions of unknown origin for some time.

Vignette 1 – Human doctor (cardiology)

You are in the examination room with a cardiologist. You tell him or her about your symptoms and a conversation about your symptoms develops. You name the following symptoms: cardiac arrhythmia, which occurs even with slight physical exertion, and shortness of breath. He or she will examine you, arrange for a resting ECG and take your blood. On the basis of a joint discussion and the ECG and laboratory results, the doctor makes the diagnosis and discusses the treatment with you.

Vignette 2 – Human doctor (orthopedics)

You are in the examination room with an orthopaedist. You tell him or her about your symptoms and a conversation about your symptoms develops. You name the following symptoms: severe back pain, which occurs particularly when sitting and standing. In addition, you tell him or her that the symptoms are more pronounced in the morning than in the evening. He or she will examine you and order an an x-ray of your spine. On the basis of a joint discussion and the x-ray findings, the doctor makes the diagnosis and discusses the treatment with you.

Vignette 3 – Human doctor (dermatology)

You are in the examination room with a dermatologist. You tell him or her about your symptoms and a conversation about your symptoms develops. You name the following symptoms: severe itching on your arms and legs that has persisted for several weeks. You also tell him or her that the scratching of the itchy areas has already caused bleeding. He or she will examine the itchy areas on your body and take a blood sample. On the basis of a joint discussion and the laboratory findings, the doctor makes the diagnosis and discusses the treatment with you.

Vignette 4 – Human doctor (psychiatry)

You are in the examination room with a psychiatrist. You tell him or her about your symptoms and a conversation about your symptoms develops. You name the following symptoms: feeling extremely listless for several months, increasing experience of anxiety, which is also accompanied by physical symptoms, in particular a racing heart, outbreaks of sweat and enormous tension and nervousness. He or she will ask you about your personal and working life. On the basis of a joint discussion, the doctor makes the diagnosis and discusses the treatment with you.

Vignette 5 – Human doctor with AI system (cardiology)

You are in the examination room with a cardiologist. You tell him or her about your symptoms and a conversation about your symptoms develops. You name the following symptoms: cardiac arrhythmia, which occurs even with slight physical exertion, and shortness of breath. He or she will examine you, arrange for a resting ECG and take your blood. A conversation with him or her follows. He or she informs you that a computer system based on artificial intelligence (AI) has automatically evaluated your reported symptoms together with the ECG and blood data, made a diagnosis and developed a treatment suggestion. The diagnosis and treatment suggestion are checked by the doctor. The doctor then discusses the diagnosis and treatment with you.

Vignette 6 – Human doctor with AI system (orthopedics)

You are in the examination room with an orthopaedist. You tell him or her about your symptoms and a conversation about your symptoms develops. You name the following symptoms: severe back pain,

which occurs particularly when sitting and standing. In addition, you tell him or her that the symptoms are more pronounced in the morning than in the evening. He or she will examine you and order an x-ray of your spine. A conversation with him or her follows. He or she informs you that a computer system that works on the basis of artificial intelligence (AI) has automatically evaluated your reported symptoms together with the X-ray images, made a diagnosis and developed a treatment suggestion. The diagnosis and treatment suggestion are checked by the doctor. The doctor then discusses the diagnosis and treatment with you.

Vignette 7 – Human doctor with AI system (dermatology)

You are in the examination room with a dermatologist. You tell him or her about your symptoms and a conversation about your symptoms develops. You name the following symptoms: severe itching on your arms and legs that has persisted for several weeks. You also tell him or her that the scratching of the itchy areas has already caused bleeding. He or she will examine the itchy areas on your body and take a blood sample. A conversation with him or her follows. He or she informs you that a computer system that works on the basis of artificial intelligence (AI) has automatically evaluated your reported symptoms together with the blood data, made a diagnosis and developed a treatment suggestion. The diagnosis and treatment suggestion are checked by the doctor. The doctor then discusses the diagnosis and treatment with you.

Vignette 8 – Human doctor with AI system (psychiatry)

You are in the examination room with a psychiatrist. You tell him or her about your symptoms and a conversation about your symptoms develops. You name the following symptoms: feeling extremely listless for several months, increasing experience of anxiety, which is also accompanied by physical symptoms, in particular a racing heart, outbreaks of sweat and enormous tension and nervousness. He or she will ask you about your personal and working life. A conversation with him or her follows. He or she informs you that a computer system that works on the basis of artificial intelligence (AI) has automatically evaluated your reported symptoms and circumstances, made a diagnosis and developed a treatment suggestion. The diagnosis and treatment suggestion are checked by the doctor. The doctor then discusses the diagnosis and treatment with you.

Vignette 9 – AI system alone (cardiology)

You are in the examination room. There is a computer system and a robot, both of which work on the basis of artificial intelligence (AI). You communicate with the computer (chatbot) via a dialog box on the screen. You enter your symptoms on the keyboard, specifically cardiac arrhythmia, which occurs even with slight physical exertion, and shortness of breath. The system asks you questions to which you answer. The robot also takes a blood sample and performs an ECG. After some time, you will be presented with a diagnosis and treatment on the computer screen, which will later be implemented by a medical team.

Vignette 10 – AI system alone (orthopedics)

You are in the examination room. There is a computer system and a robot, both of which work on the basis of artificial intelligence (AI). You communicate with the computer (chatbot) via a dialog box on the screen. You enter your symptoms on the keyboard, specifically severe back pain, which occurs particularly when sitting and standing. In addition, you communicate that the symptoms are more pronounced in the morning than in the evening. The system asks you questions to which you answer.

An x-ray is also initiated. After some time, you will be presented with a diagnosis and treatment on the computer screen, which will later be implemented by a medical team.

Vignette 11 – AI system alone (dermatology)

You are in the examination room. There is a computer system and a robot, both of which work on the basis of artificial intelligence (AI). You communicate with the computer (chatbot) via a dialog box on the screen. You enter your symptoms on the keyboard, specifically severe itching on your arms and legs that has persisted for several weeks. You also communicate that the scratching of the itchy areas has already caused bleeding. The system asks you questions, which you answer. The robot also takes a blood sample. After some time, you will be presented with a diagnosis and treatment on the computer screen, which will later be implemented by a medical team.

Vignette 12 – AI system alone (psychiatry)

You are in the examination room. There is a computer system and a robot, both of which work on the basis of artificial intelligence (AI). You communicate with the computer (chatbot) via a dialog box on the screen. You enter your symptoms on the keyboard, specifically that you have been feeling extremely listless for several months and are increasingly experiencing anxiety, which is also accompanied by physical symptoms, in particular a racing heart, outbreaks of sweat and enormous tension and nervousness. The system asks you questions about your circumstances in relation to your private and work life, to which you answer. After some time, you will be presented with a diagnosis and treatment on the computer screen, which will later be implemented by a medical team.

Appendix B

This appendix summarizes the measurement instruments of the latent constructs, including all items and Cronbach's Alphas (α).

Table 1 Appendix B. Measurement instruments and Cronbach's Alphas (**α)**

**Latent construct and items**	**α or correlation between items**
Trust (Soellner et al. 2012)<br><br>• The diagnosis decision is trustworthy.<br>• The treatment decision is trustworthy.<br>• I have a good feeling when relying on the diagnosis decision.<br>• I have a good feeling when relying on the treatment decision.<br>• I trust the information presented by the [physician / physician who is supported by the chatbot / chatbot]	.97
Distrust (Soellner et al. 2012, reverse formulation of trust items)<br><br>• The diagnosis decision is untrustworthy.<br>• The treatment decision is untrustworthy.<br>• I have a bad feeling when relying on the diagnosis decision.<br>• I have a bad feeling when relying on the treatment decision.<br>• I distrust the information presented by the [physician / physician who is supported by the chatbot / chatbot]	.97
Privacy invasion (Fischer et al. 2021)<br><br>• I fear that my interaction with the [physician / physician who is supported by the chatbot / chatbot] is less confidential than I would like it to be.<br>• I fear that the information that I exchange with the [physician / physician who is supported by the chatbot / chatbot] is not as protected as I would like it to be.<br>• My personal health information is too easily accessible due to my interaction with the [physician / physician who is supported by the chatbot / chatbot].<br>• I fear that my personal data can easily be stolen as a consequence of my interaction with the [physician / physician who is supported by the chatbot / chatbot].	.98

Information disclosure (Bansal et al. 2010)<br><br>• I would be very likely to disclose health information to the [physician / physician who is supported by the chatbot / chatbot].<br>• I have a strong intention to disclose health information to the [physician / physician who is supported by the chatbot / chatbot].<br>• I would definitely disclose health information to the [physician / physician who is supported by the chatbot / chatbot].	.97
Treatment adherence (DiMatteo et al. 1992)<br><br>• I will have a hard time doing what the [physician / physician who is supported by the chatbot / chatbot] suggested that I should do. (reverse coded)<br>• I will follow the [physician's / chatbot supported physician's / chatbot's] suggestions exactly.<br>• I will be unable to do what will be necessary to follow the [physician's / chatbot supported physician's / chatbot's] treatment plans. (reverse coded)<br>• I will find it easy to do the things the [physician / physician who is supported by the chatbot / chatbot] suggested that I should do.	.94
Satisfaction (Probst et al. 1997)<br><br>• This type of diagnosis and treatment development method seems to pay attention as I describe my condition.<br>• This type of diagnosis and treatment development method handles me with care during the examination.<br>• This type of diagnosis and treatment development method makes me feel as if I could talk about any type of problem.<br>• In general, I would be satisfied with this type of diagnosis and treatment development method.	.96
Personality (based on Gosling et al. 2003)<br><br>Extraversion<br>• I am rather reserved, cautious. (reverse coded)<br>• I come out of myself, am sociable.<br><br>Agreeableness<br>• I easily give others trust, believe in the good in people.<br>• I tend to criticize others. (reverse coded)<br><br>Conscientiousness<br>• I make myself comfortable, tend toward laziness. (reverse coded)<br>• I complete tasks thoroughly.	Correlations between items of each scale:<br><br>E: r = .51<br>p < .001<br><br>A: r = .07<br>p = .012<br><br>C: r = .20<br>p < .001

Neuroticism<br><br>• I am relaxed, do not let stress unsettle me. (reverse coded)<br>• I easily get nervous and insecure.<br><br>Openness<br><br>• I have little artistic interest. (reverse coded)<br>• I have an active imagination.	N: r = .43<br>p < .001<br><br>O: r = .32<br>p < .001
Disposition to trust humans (Gefen 2000)<br><br>• I generally trust other people.<br>• I tend to count upon other people.<br>• I generally have faith in humanity.<br>• I generally trust other people unless they give me reason not to.	.81
Disposition to trust machines (Gefen 2000, adjusted formulation of disposition to trust humans items)<br><br>• I generally trust machines.<br>• I tend to count upon machines.<br>• I generally have faith in the effective operation of machines.<br>• I generally trust machines unless they give me reason not to.	.78
Technology attitude (Nam 2019). This construct was measured with two sub-dimensions: enthusiasm and worry.<br><br>Enthusiasm<br><br>Thinking about the possibility that computers and robots could do most of the work currently done by humans, how enthusiastic are you, if at all, about this possibility for society as a whole?<br><br>• not at all enthusiastic<br>• not too enthusiastic<br>• somewhat enthusiastic<br>• very enthusiastic	Correlation between items:<br><br>$r = -.29$<br><br>$p < .001$

Worry<br><br>Thinking about the possibility that computers and robots could do most of the work currently done by humans, how worried are you, if at all, about this possibility for society as a whole?<br><br>• not at all worried<br>• not too worried<br>• somewhat worried<br>• very worried	
AI phobia (Khasanwneh 2018)<br><br>• I am fearful that someone is using technology to watch and listen to everything that I do.<br>• I am terrified that technologies will change the way we live, communicate, love, and even judge others.<br>• I am afraid of new technologies because one day they will make us (humans) obsolete.<br>• I am fearful that new technologies will someday take over my job.<br>• I am afraid of new technologies because they may interfere with my life emotionally, physically, and psychologically.<br>• I am afraid to use some features in my cell phone.<br>• I am terrified of being connected to the Internet, because someone might be tracking me.	.85

Note: All latent constructs except technology attitude (Nam 2019) were measured with a 5-point scale, where participants expressed their agreement with each item. The scale ranged from 1 "not at all correct" to 5 "completely correct".

Appendix C

This appendix shows the correlation table with the six main factors investigated.

		Trust	Distrust	Privacy Invasion	Information Disclosure	Treatment Adherence	Satisfaction
Trust	r	1					
	p						
Distrust	r	-.698	1				
	p	.000					
Privacy Invasion	r	-.459	.707	1			
	p	.000	.000				
Information Disclosure	r	.829	-.588	-.535	1		
	p	.000	.000	.000			
Treatment Adherence	r	.779	-.802	-.620	.734	1	
	p	.000	.000	.000	.000		
Satisfaction	r	.936	-.637	-.432	.843	.748	1
	p	.000	.000	.000	.000	.000	

Note: All correlations (r) are significant at $p < 0.01$ (two-tailed).

Appendix D

This appendix presents the means and standard errors of the means (SEM) for each of the six main factors investigated.

Trust

Interaction partner	Medical discipline	Mean	SEM
Human doctor	Cardiology	3.885	.024
	Orthopaedics	3.894	.024
	Dermatology	3.874	.024
	Psychiatry	3.812	.025
Human doctor with AI	Cardiology	3.572	.027
	Orthopaedics	3.563	.027
	Dermatology	3.580	.027
	Psychiatry	3.348	.029
AI system only	Cardiology	2.708	.030
	Orthopaedics	2.739	.029
	Dermatology	2.727	.029
	Psychiatry	2.642	.030

Distrust

Interaction partner	Medical discipline	Mean	SEM
Human doctor	Cardiology	2.077	.026
	Orthopaedics	2.079	.026
	Dermatology	2.091	.026
	Psychiatry	2.154	.026
Human doctor with AI	Cardiology	2.394	.028
	Orthopaedics	2.420	.029
	Dermatology	2.411	.028
	Psychiatry	2.618	.029
AI system only	Cardiology	3.151	.030
	Orthopaedics	3.151	.029
	Dermatology	3.187	.029
	Psychiatry	3.252	.030

Privacy Invasion

Interaction partner	Medical discipline	Mean	SEM
Human doctor	Cardiology	2.261	.028
	Orthopaedics	2.292	.028
	Dermatology	2.282	.028
	Psychiatry	2.301	.029
Human doctor with AI	Cardiology	2.722	.030
	Orthopaedics	2.711	.030
	Dermatology	2.740	.030
	Psychiatry	2.786	.030
AI system only	Cardiology	3.031	.032
	Orthopaedics	3.014	.032
	Dermatology	3.005	.032
	Psychiatry	3.042	.032

Information Disclosure

Interaction partner	Medical discipline	Mean	SEM
Human doctor	Cardiology	3.820	.026
	Orthopaedics	3.818	.025
	Dermatology	3.797	.025
	Psychiatry	3.756	.026
Human doctor with AI	Cardiology	3.510	.027
	Orthopaedics	3.488	.027
	Dermatology	3.482	.027
	Psychiatry	3.348	.028
AI system only	Cardiology	2.931	.031
	Orthopaedics	2.975	.030
	Dermatology	2.947	.030
	Psychiatry	2.869	.031

Treatment Adherence

Interaction partner	Medical discipline	Mean	SEM
Human doctor	Cardiology	3.714	.022
	Orthopaedics	3.710	.022
	Dermatology	3.704	.022
	Psychiatry	3.637	.022
Human doctor with AI	Cardiology	3.472	.023
	Orthopaedics	3.464	.023
	Dermatology	3.499	.023
	Psychiatry	3.334	.023
AI system only	Cardiology	2.980	.024
	Orthopaedics	3.007	.024
	Dermatology	3.016	.024
	Psychiatry	2.939	.024

Satisfaction

Interaction partner	Medical discipline	Mean	SEM
Human doctor	Cardiology	3.868	.025
	Orthopaedics	3.866	.024
	Dermatology	3.870	.024
	Psychiatry	3.816	.024
Human doctor with AI	Cardiology	3.560	.027
	Orthopaedics	3.551	.027
	Dermatology	3.556	.027
	Psychiatry	3.306	.030
AI system only	Cardiology	2.660	.031
	Orthopaedics	2.686	.030
	Dermatology	2.657	.030
	Psychiatry	2.600	.031

Appendix E

In this appendix we present the results of our calculations regarding the control variables. The covariates were z-transformed before being entered in the ANCOVA models. For each control variable a separate ANCOVA model was computed. The list of the control variables is as follows: personality (extraversion, neuroticism, openness, agreeableness, and conscientiousness), disposition to trust humans, disposition to trust machines, AI phobia, and technology attitude (enthusiasm and worry). Further categorial control variables (chronic illness, regular medication intake, sex, education and age-group) were tested in separate additional ANOVA models.

With respect to the control variables which we entered as covariates into the ANCOVA models (personality with its dimensions extraversion, neuroticism, openness, agreeableness, and conscientiousness; disposition to trust humans; disposition to trust machines; AI phobia; and technology attitude with its dimensions enthusiasm and worry), we see in part substantial effects on the six major outcome variables (see Table 1 Appendix E, upper part). However, all covariate main or interaction effects are less strong than the effects of the patient's interaction partner. Moreover, these effects were mostly expected and hence are not surprising. For example, disposition to trust machines explains a substantial amount of variance in trust (η_p^2 = .167).

Additional ANOVA models including chronic illness, regular medication intake, sex, education, and age group as additional between-subject factors revealed occasional main or interaction effects; however, these effects were marginal in comparison to the effects of the patient's interaction partner and medical discipline (i.e., the two manipulated factors; see Table 1 Appendix E, lower part).

Table 1 Appendix E. Presentation of the strongest effects of control variables in terms of explained variance (partial eta^2)

	Trust	Distrust	Privacy Invasion	Information Disclosure	Treatment Adherence	Satisfaction
Covariates						
Extraversion	.010[§]	.009[§]	.002[§]	.010[§]	.010[§]	.013[§]
Neuroticism	.008[§]	.010[§]	.008[§]	.005[§]	.008[§]	.014[§]
Openness	.007[$]	.016[§]	.008[$]	.011[§]	.029[§]	.010[$]
Agreeableness	.023[§]	.019[§]	.010[§]	.017[§]	.014[§]	.022[§]
Conscientiousness	.015[$]	.026[§]	.012[$]	.019[$]	.028[§]	.019[$]
Disposition to Trust humans	.066[§]	.010[§]	.006[§]	.054[§]	.021[§]	.054[§]
Disposition to trust machines	.167[§]	.055[§]	.049[§]	.148[§]	.105[§]	.157[§]
AI phobia	.065[§]	.176[§]	.209[§]	.077[§]	.137[§]	.052[§]
Technology attitude: Enthusiasm	.081[§]	.039[$]	.030[$]	.079[§]	.049[$]	.074[§]
Technology attitude: Worry	.073[§]	.067[§]	.073[§]	.069[§]	.070[§]	.062[§]
Between-subject factors						
Chronic illness	.003[*]	.001[$#*]	.001[#]	.001[%#]	.003[$#]	.001[$%#*]
Regular medication intake	.004[%]	.004[$]	.002[$]	.003[$]	.006[$]	.003[$%#*]
Sex	.009[$]	.009[$]	.007[$]	.007[$]	.013[$]	.015[$]
Education	.011[*]	.014[*]	.014*	.019*	.018*	.016[$]
Age group	.005[$%]	.005[$*]	.007[$]	.008[$]	.007[$]	.007[$]

Note. All covariates were z-transformed before their inclusion in the model. [§] Main effect covariate, [*] main effect between-subject factor, [$] interaction with patient's interaction partner, [%] interaction with medical discipline, [#] interaction with patient's interaction partner x medical discipline.

Appendix F

In this appendix we present our LISREL model. In this model all measurement items—lambda-X (LX) and lambda-Y (LY)—of trust (TR, 12 items), distrust (DI, 12 items), and privacy invasion (PI, 12 items) (formally expressed as TR/DI/PI_LXij with i = number of patient's interaction partner (i = 1, 2, 3) and j = number of medical discipline (j = 1, 2, 3, 4)), as well as all measurement items of information disclosure (ID, 3 items), treatment adherence (TA, 4 items), and satisfaction (SA, 4 items) (formally expressed as ID/TA/SA_LYij with i = number of patient's interaction partner (i = 1, 2, 3) and j = number of medical discipline (j = 1, 2, 3, 4)), represent the observed variables. Furthermore, the mediators (trust, distrust, privacy invasion) (Ksi formally expressed as $\xi1$, $\xi2$, $\xi3$), as well as the dependent variables (information disclosure, treatment adherence, satisfaction) (Eta formally expressed as $\eta1$, $\eta2$, $\eta3$), represent the latent variables. In our model, we specify: $\xi1$ = TR, $\xi2$ = DI, $\xi3$ = PI; $\eta1$ = ID, $\eta2$ = TA, $\eta3$ = SA; i1 = human doctor, i2 = human doctor with AI system, i3 = AI system only; j1 = cardiology, j2 = orthopedics, j3 = dermatology, j4 = psychiatry. The statistical foundations of our approach can be found in Jöreskog (1970). In the following Results section, we present a graphical representation of our LISREL model with the corresponding statistics.

Figure 1 Appendix F shows a conceptual representation of our LISREL model. The corresponding empirical path diagram is shown in Figure 2 Appendix F; the corresponding standardized path coefficients are summarized in Tables 1 and 2 Appendix F. Because both distrust and privacy invasion do not have a statistically significant influence on satisfaction (see Table 2 in the main manuscript), the corresponding paths (i.e., DI and PI to SA) are not shown. Regarding specification of the LISREL model, it can be seen that the six variables (trust, distrust, privacy invasion, information disclosure, treatment adherence, satisfaction) are conceptualized as reflective constructs.[1]

[1] Thus, the indicators (i.e., the items in the questionnaire) are caused by the latent variables (i.e., trust, distrust, privacy invasion, information disclosure, treatment adherence, satisfaction).

Figure 1 Appendix F. Conceptual Representation of the LISREL Model

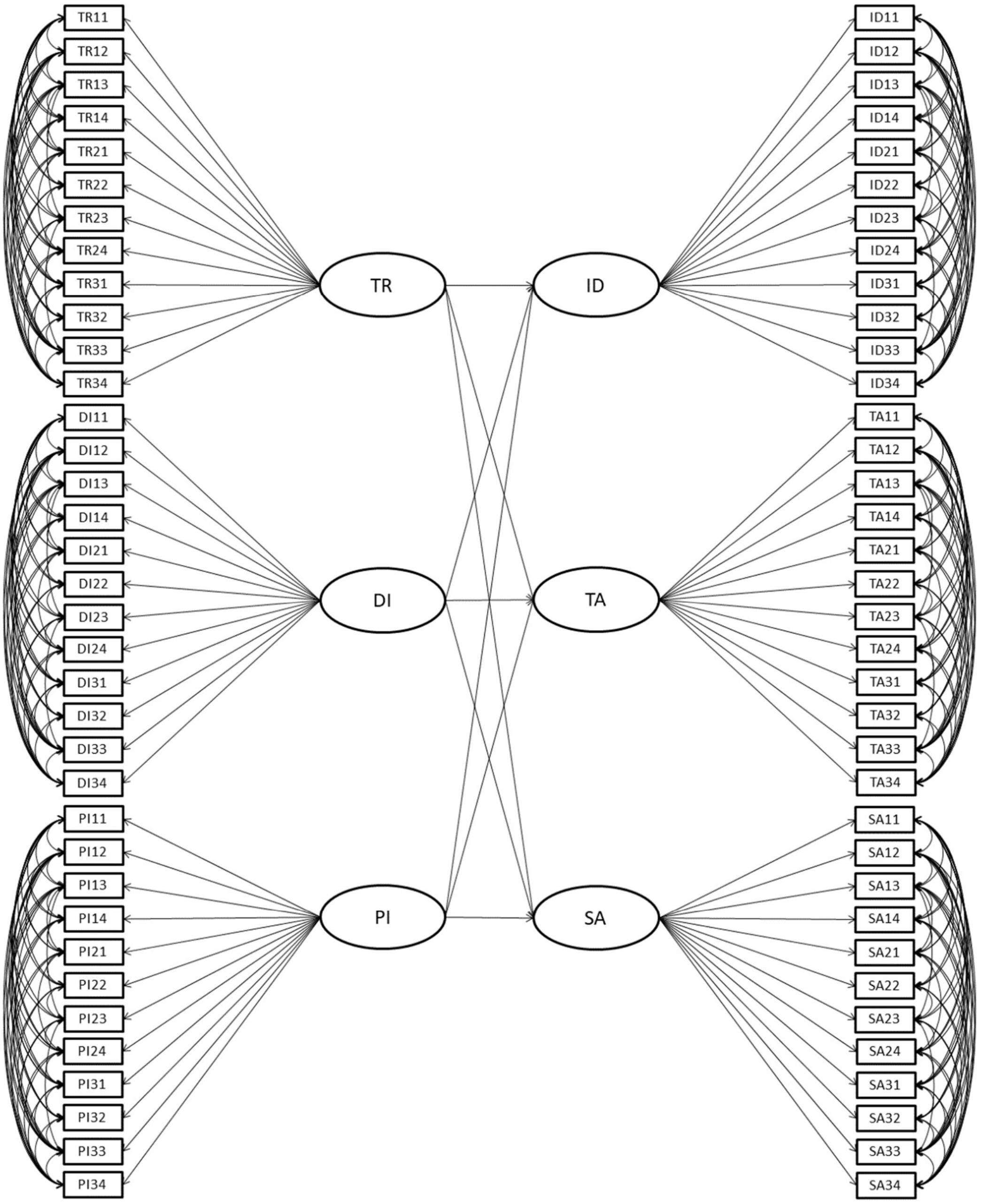

Note: TR = Trust, DI = Distrust, PI = Privacy Invasion, ID = Information Disclosure, TA = Treatment Adherence, SA = Satisfaction.

Figure 2 Appendix F. Empirical Path Diagram

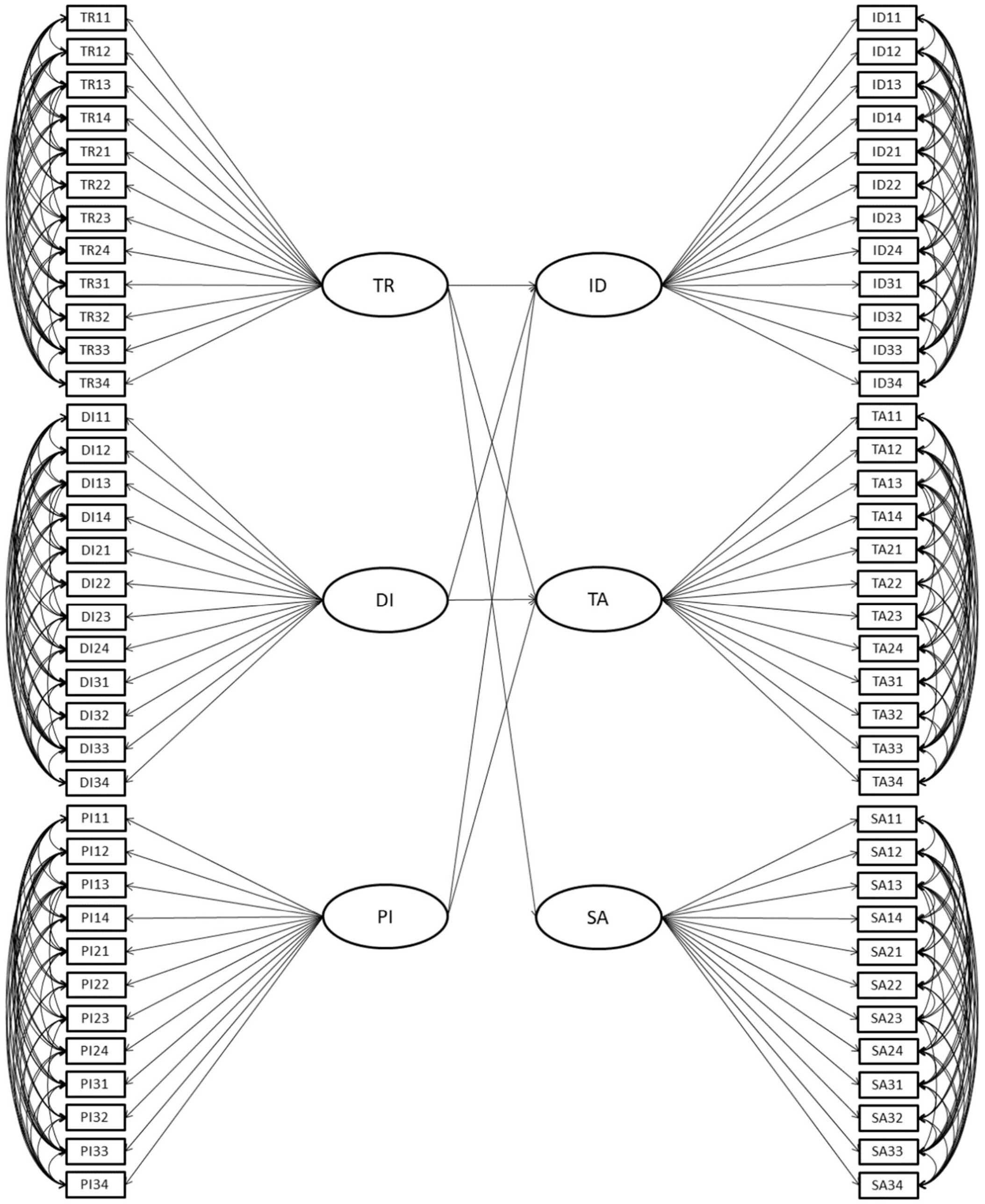

Note: TR = Trust, DI = Distrust, PI = Privacy Invasion, ID = Information Disclosure, TA = Treatment Adherence, SA = Satisfaction.

In the following, we indicate the standardized path coefficients of the LISREL model as shown in Figure 2 Appendix F.

Table 1 Appendix F. Values of lambda-X

	TR	DI	PI
TR11	0.41		
TR12	0.39		
TR13	0.41		
TR14	0.40		
TR21	0.61		
TR22	0.61		
TR23	0.61		
TR24	0.56		
TR31	0.35		
TR32	0.36		
TR33	0.34		
TR34	0.32		
DI11		0.40	
DI12		0.37	
DI13		0.42	
DI14		0.40	
DI21		0.59	
DI22		0.61	
DI23		0.60	
DI24		0.56	
DI31		0.33	
DI32		0.35	
DI33		0.34	
DI34		0.32	
PI11			0.43
PI12			0.42
PI13			0.42
PI14			0.43
PI21			0.65
PI22			0.66
PI23			0.66
PI24			0.65
PI31			0.55
PI32			0.54
PI33			0.54
PI34			0.53

Note: TR = Trust, DI = Distrust, PI = Privacy Invasion.

Table 2 Appendix F. Values of lambda-Y

	ID	TA	SA
ID11	0.35		
ID12	0.43		
ID13	0.45		
ID14	0.35		
ID21	0.64		
ID22	0.64		
ID23	0.66		
ID24	0.62		
ID31	0.43		
ID32	0.43		
ID33	0.44		
ID34	0.42		
TA11		0.25	
TA12		0.38	
TA13		0.37	
TA14		0.37	
TA21		0.58	
TA22		0.57	
TA23		0.57	
TA24		0.54	
TA31		0.38	
TA32		0.38	
TA33		0.39	
TA34		0.35	
SA11			0.37
SA12			0.36
SA13			0.37
SA14			0.37
SA21			0.57
SA22			0.56
SA23			0.56
SA24			0.49
SA31			0.29
SA32			0.29
SA33			0.27
SA34			0.26

Note: ID = Information Disclosure, TA = Treatment Adherence, SA = Satisfaction.
